# The Audiphone and Dentiphone

**Published:** 1880-03

**Authors:** 


### ARTICLE V.

### *The Audiphone and Dentaphone.*

Dr. Chas. S. Turnbull, of Philadelphia, describes these instruments, in the *Archives of Otology*, as follows:

"The 'audiphone' consists essentially of a diaphragm of hard rubber. This diaphragm is very thin and elastic, and is cut in the form of a square with rounded corners, so as to present a collecting surface about one square foot in size. For purposes of convenient adjustment, it is furnished with neat hard rubber handle, and might easily, says the inventor, be taken for a fan of Japanese pattern. When in use the upper and lower edges are made to approximate by a silken cord, so as to present a convex surface to the speaker and a concave one to the listener. The cord may be fastened at any convenient convexity of the surface of the auditory disk. When adjusted, the upper edge is pressing firmly against the anterior surface of the upper incisors, allowing the upper lip to rest upon the diaphragm, and the deaf person is then ready to listen." If the eye teeth can be used, they generally give the best results. False teeth may also be used, especially if they fit tightly; should they.not, however, they may be made to do so by pressing the lower teeth against them. If the natural teeth be too far gone to be used as directed, the roots may in many instances be utilized by having artificial teeth set into them. The handle of the audiphone should be held lightly, and the lower teeth should not touch the diaphragm, nor should it be held between the teeth or pressed too forcibly against the upper ones, thus curving the instrument already bent by the cord. It must be borne in mind that in all cases the vibrations of the upper edge of the disk impart to the upper teeth the sound waves, which are transmitted through them to the cranial bones and auditory nerves.

The audiphone, therefore, is entirely dependent upon the condition of the auditory nerves, because in direct proportion to the inherent power of these nerves—independent of the external and middle ears or acoustic apparatus—is the influence which this and all similar appliances will exert over the hearing power.

Absolute nerve deafness, which is comparatively rare, is in no way whatever benefitted by the application of the audiphone.

This deafness is caused by direct application of the auditory nerve, through malignant, scarlet and typhoid fevers, cerebro-spinal and other forms of meningitis, tertiary syphilis, cerebral tumors, trauma, consanguinity, hereditation, old age, etc.

Profound acoustic deafness, which is likewise comparatively rare, is markedly, and in some cases signally, relieved by the use of the audiphone.

This deafness is caused through direct implication of the middle ear (or conducting apparatus) and its appendages, through the several forms of catarrhal and purulent inflammations, scarlet and typhoid fevers, secondary syphilis, trauma, consanguinity, hereditation, old age, etc.

Those who are *partially* deaf, from whatever cause, as a rule, derive no benefit from the application of the audiphone; on the contrary, many such cases are annoyed by hyperacusis, etc.

Therefore, the number of cases in which "the deaf are made to hear" with the audiphone is comparatively small, when we take into consideration the whole number of our deaf population. Audition will be improved by its use in but few of the many deaf persons who enlist the service of an aurist.

To use the audiphone with success the auditory nerves must be normally sensitive, the hearing power for loud voice, through middle ear deafness, must be reduced to a minimum, and the upper front teeth must be solid. The acoumeter, the tuning fork, a thin sheet of vulcanite, of iron, of ash or poplar wood, and best of all, a sheet of bristol board or sized paper, will in every case enable us to decide whether the audiphone or its principal can be successfully applied.

Concerning *absolutely deaf mutes* and the audiphone, we need say nothing further. They must be left to the patient teachers of the several methods of educating the true deaf mutes.

To the *semi-deaf mutes*, however, the audiphone will open a new world of enjoyment, and prove a useful instrument, especially in the hands of all instructors in our asylums for the deaf and dumb, in educating children according to Bell's method of visible speech; especially as very few even of those who are supposed to be born deaf are totally without some slight degree of hearing power; hence, nearly all of those educated in the asylums may be taught to speak, inasmuch as their dumbness is owing solely to their want of use of the organs of speech. "Mutes," says the inventor, "will learn to speak by holding the audiphone against the teeth, as already directed, and practicing speaking while it is in this position." It is a good exercise for the mute, at first, to put one hand on the instructor's throat, watch the motion of his lips, while his other hand is on his own throat, the instructor meantime holding the audiphone to the mute's teeth. The mute will *feel* the influence of the sound in his hand on the instructor's throat, imitate it in his own throat, will *hear* the speaker's voice on the audiphone, and will be aided in imitating the speaker by *seeing* his lips, and will also *hear* his own phonation sounds as reflected from the audiphone, and the more readily, therefore, learn to articulate.

Music, its varying sounds and harmonies, as conveyed by means of the audiphone, awakens in the semi-deaf mutes an unusually pleasurable sensation, as manifested by their gesticulations and facial expression.

Under the pretext of being a fan, the instrument can seldom be used, and, being cumbersome and conspicuous, is open to the same objections as the ear-trumpet.

The "Dentaphone" is a naval instrument of the same practical application and acoustic principle as the audiphone, but constructed more after the plan of the telephone, and it "consists, in brief, of a chambered box (similar to a telephonic mouth-piece) in which is secured an exceedingly delicate, easily vibrating, diaphragm. Connecting this with a wooden tooth-piece is a silken cord of variable length. The person using the dentaphone simply holds the instrument receiver in his hand, in any convenient position, with the tooth-piece between the teeth, and the open side of the receiver facing toward the speaker. The silk conducting line connecting the receiver with the tooth-piece should be kept moderately tight, and may be shortened or lengthened to suit the convenience of the person using the instrument."

The dentaphone weighs but one ounce and a half, and can easily be carried about the person. In testing the instrument, it compares most favorably with the audiphone, and answers fully as well for all requirements.

It is used for precisely the same class of cases as are improved by the audiphone, and bids fair to be a powerful rival.

Concerning an appropriate or descriptive name, we would prefer the term "Dento-Audiphone," and recommend the substitution of fans (made of thin, elastic wood, "bristol" or "binder's boards," which are to be held between or against the teeth, and bent into a curve by pressure from the handle toward the teeth.—*Med. and Surg. Reporter*.

---

